# Significance of G Protein-Coupled Estrogen Receptor in the Pathophysiology of Irritable Bowel Syndrome, Inflammatory Bowel Diseases and Colorectal Cancer

**DOI:** 10.3389/fendo.2020.00390

**Published:** 2020-06-12

**Authors:** Damian Jacenik, Wanda M. Krajewska

**Affiliations:** Department of Cytobiochemistry, Faculty of Biology and Environmental Protection, University of Lodz, Lodz, Poland

**Keywords:** G protein-coupled estrogen receptor, irritable bowel syndrome, inflammatory bowel diseases, Crohn's disease, ulcerative colitis, colorectal cancer

## Abstract

The regulatory role of estrogens and nuclear estrogen receptors, i. e., estrogen receptor α and β has been reported in gastrointestinal diseases. However, the contribution of G protein-coupled estrogen receptor, the membrane-bound estrogen receptor, is still poorly understood. Unlike nuclear estrogen receptors, which are responsible for the genomic activity of estrogens, the G protein-coupled estrogen receptor affects the “rapid” non-genomic activity of estrogens, leading to modulation of many signaling pathways and ultimately changing gene expression. Recently, the crucial role of G protein-coupled estrogen receptor in intestinal pathogenesis has been documented. It has been shown that the G protein-coupled estrogen receptor can modulate the progression of irritable bowel syndrome, inflammatory bowel diseases such as Crohn's disease and ulcerative colitis as well as colorectal cancer. The G protein-coupled estrogen receptor appears to be a potent factor regulating abdominal sensitivity and pain, intestinal peristalsis, colitis development, proliferation and migration potential of colorectal cancer cells and seems to be a useful target in gastrointestinal diseases. In this review, we present the current state of knowledge about the contribution of the G protein-coupled estrogen receptor to irritable bowel syndrome, inflammatory bowel diseases and colorectal cancer.

## Introduction

**Figure 1:**
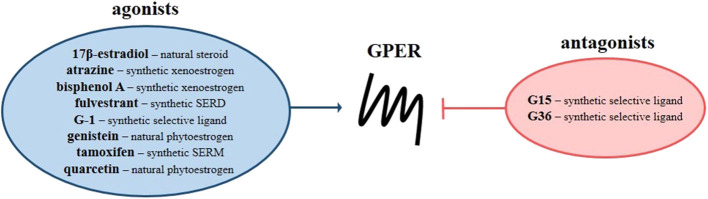
G protein-coupled estrogen receptor ligands. Scheme presents natural and synthetic agonists (blue circle) and antagonists (red circle) of GPER. SERD, selective nuclear estrogen receptor down-regulators; SERM, selective nuclear estrogen receptor modulators.

The G protein-coupled estrogen receptor (GPER, previously known as GPR30) is a seven-transmembrane receptor discovered, among others, in breast cancer tissue and estrogen receptor-positive MCF-7 cell line (1–7). In addition to acting *via* nuclear estrogen receptors (ERs), i.e., ERα and ERβ, estrogens have been reported to induce ligand-dependent signaling by the membrane-bound estrogen receptor named GPER. In contrast to nuclear ERs, which predominantly regulate expression of target genes through direct interaction with estrogen response element or indirectly through transcription factors, GPER is responsible for “rapid” non-genomic activity of estrogens, leading to modulation of many signaling pathways and ultimately gene expression. Studies of the mechanisms underlying the effects of GPER under physiological and pathological conditions have shown that activation of GPER leads to stimulation of signaling pathways dependent on both Gα_s_ and Gβγ proteins. Epidermal growth factor receptor (EGFR), mitogen-activated protein kinases (MAPKs), phosphatidylinositol 3- kinase (PI3K), phospholipase C (PLC) and adenylyl cyclase (AC) are the main GPER-dependent pathways regulated by the action of G proteins (8–12). It was also found that GPER is capable to affect nuclear factor-κB (NF-κB) and Notch as well as Hippo signaling, where the membrane-bound estrogen receptor regulates phosphorylation of crucial proteins through Gα_q−11_ action, enhancing the proliferation and migration potential of breast cancer cells (11, 13, 14). GPER action appears unrelated to G protein-independent signals such as β-arrestin recruitment (15, 16). Beyond 17β-estradiol, which is a natural agonist of GPER, various ligands have an affinity for this estrogen receptor (Figure 1). Among them there are therapeutic agents that belong to the classes of selective nuclear ER modulators (e.g., tamoxifen), selective nuclear ER down-regulators (e.g., fulvestrant), and xenoestrogens (e.g., atrazine, bisphenol A, genistein and quarcetin) as well as synthetic GPER selective agonist (i.e., G-1) and antagonists (i.e., G15 and G36) (17). All synthetic GPER selective ligands are based on the tetrahydro-3H-cyclopenta[c]quinoline scaffold, but the GPER antagonist G15 is characterized by the absence of ethanone moiety compared to the GPER agonist G-1, while the second GPER antagonist, i.e., G36, has an isopropyl moiety in the place of the ethanone moiety at position C8.

GPER expression is not restricted to estrogen responsive tissues. GPER is widely expressed in human tissues, including breast, ovaries, uterus, placenta, testis, prostate, bone marrow, thymus, bones, smooth and skeletal muscles, brain, blood vessels, heart, lung, liver and intestine (18). Considerable evidence suggests that GPER is essential in cardiovascular diseases, obesity, diabetes, immune disorders and infectious diseases as well as in neoplastic transformation and tumor progression (17, 19, 20). In this review, we summarize the evidence for GPER expression and function in the pathophysiology of intestinal diseases, i.e., irritable bowel syndrome, inflammatory bowel diseases and colorectal cancer.

## Irritable Bowel Syndrome

Irritable bowel syndrome (IBS) is a common, functional gastrointestinal disorder that is manifested by abdominal pain and bowel habit disturbance. From a clinical point of view, IBS is divided into subtypes related to changes in gastrointestinal motility, i.e., diarrhea-predominant IBS (IBS-D), constipation-predominant IBS (IBS-C) and IBS with altered bowel habits (IBS-A or IBS-M). The pathophysiology of IBS is still elusive, but several factors such as microbiota, environmental and genetic variations, seem to be responsible for the development of IBS.

The first evidence suggesting the importance of GPER in IBS was revealed by Qin et al. (21) who evaluated the expression of estrogen receptor genes in the intestine of IBS patients. Higher expression of GPER at mRNA level in the intestine of IBS-D patients in relation to IBS-C patients and healthy subjects was documented. Jacenik et al. (22) also observed overexpression of GPER at the level of mRNA in colonic tissue, but in both IBS-C and IBS-D patients. However, after taking into account the sex and age of the patients, statistically significant overexpression of GPER at mRNA level was observed only in men with IBS-D, suggesting a gander-specific role of estrogen signaling through this estrogen receptor in the progression of functional bowel diseases.

There are several hypotheses regarding the mechanisms by which GPER may be engaged in the progression of IBS, one of which is related with mast cell regulation. Mast cells are one of the main cell types involved in the activation of immune response in the gastrointestinal tract. Stimulated mast cells degranulate and release a wide spectrum of mediators such as amines, proteoglycans, proteases and lysosomal enzymes as well as cytokines, which leads to modulation of permeability and regulation of smooth muscle contraction (23). In IBS, alteration of mast cells number or density in the intestine was documented in several independent studies and seems to be associated with IBS pathogenesis (24–26). Although the results of Sundin et al. (27) indicate that there is no change in the infiltration and localization of mast cells in the colonic mucosa of IBS patients compared to healthy individuals, they report evidence indicating a relationship between abdominal sensitivity and mast cell number. GPER seems to be expressed in the tryptase^+^ mast cells and the expression of GPER in the cytoplasm of mast cells and GPER^+^ cells was found to be significantly higher in the colonic mucosa of IBS-D patients compared to IBS-C patients and healthy subjects. It should be noted that Qin et al. (21) found a positive correlation between the number of GPER^+^ cells and the severity of abdominal pain and not the duration of symptoms in the IBS-D patients. The importance of GPER activity modulation on abdominal pain was confirmed in a mouse model. Zielinska et al. (28) found that treatment with GPER agonists, i.e., 17β-estradiol or G-1, is associated with lower pain-induced behaviors in mice treated with mustard oil. In contrast, GPER selective antagonist G15 reduces the positive effect of GPER agonists on pain-induced behaviors.

*In vivo* studies in which Xu et al. (29) used control, stressed, and ovariectomized (OVX) rats revealed that estrogen receptor ligands acting through GPER are able to regulate visceral hypersensitivity, mast cell degranulation and mast cell tryptase expression as well as histamine levels in rat intestine. It was estimated that rats subjected to wrap partial restraint stress were characterized by increased visceromotor response. In the intestine of stressed rats a higher number of mast cells and up-regulated level of histamine were found. Both effects were reduced when OVX rats were used compared to control rats. Xu et al. (29) observed that the administration of 17β-estradiol led to an increase of visceromotor response, but pre-treatment with the GPER selective antagonist G15 counteracted the enhancing effects in OVX rats. Consistent with this, OVX rats treated with GPER selective agonist G-1 manifested dose-dependent up-regulated visceromotor response levels. It should be noted that in addition to modulating visceral motor response, GPER ligands also affect the expression level of tryptase in mast cells and histamine in the rat intestines, indicating the important role of GPER and mast cells in colon hypersensitivity in the female IBS rat model (29).

Mast cells appear not to be the only cell type involved IBS development. Enteric neurons and enteric glial cells play an essential role in the regulation of gastrointestinal motility, and motility impairment is the main hallmark of functional gastrointestinal diseases, including IBS. It has to be noted that changes in gastrointestinal motility are primarily driven by alteration of enteric nervous system, i.e., complex network of enteric neurons and glia which regulate for instance fluid exchange across the mucosa, blood flow in the intestine and gastrointestinal motility (30, 31). GPER has been shown to be expressed in the neuronal population of cells in the human and mouse intestines (29, 32, 33). Liu et al. (33) by immunofluorescent staining documented that GPER is present in the cytoplasm of enteric neurons and glial cells of the stomach, duodenum, jejunum, ileum and colon of male and female mice.

Two independent studies indicate that modulation of GPER activity affects colonic motility involving both neuronal cells and circular muscle strip contraction (28, 32). It was found that GPER inhibition with the GPER selective antagonist G15 decreases colonic transit time in the proestrus and estrus phases but not diestrus stage compared to untreated phase-matched female mice. It has been proven that 17β-estradiol administration prolonged the colonic transit time while G15 treatment reduced the effect of exogenous estrogen on colonic transit time in OVX mice. *Ex vivo* analysis using colonic circular muscle strips confirmed that GPER activation with the GPER selective agonist G-1 reduced the contractile response of the muscle strips to carbachol and this phenomenon was abolished by tetrodotoxin, suggesting that GPER may act through a neurogenic mechanism. In fact, it has been documented that GPER activation stimulated the release of nitric oxide in myenteric neurons, which was decreased by the nitric oxide synthase inhibitor (32). *In vivo* experiments using colonic bead expulsion test and mouse model of hypermotility carried out by Li et al. (32) and Zielinska et al. (28) have documented that GPER activation prolongs colonic transit time and is associated with lower number of fecal pellets.

**Figure 2:**
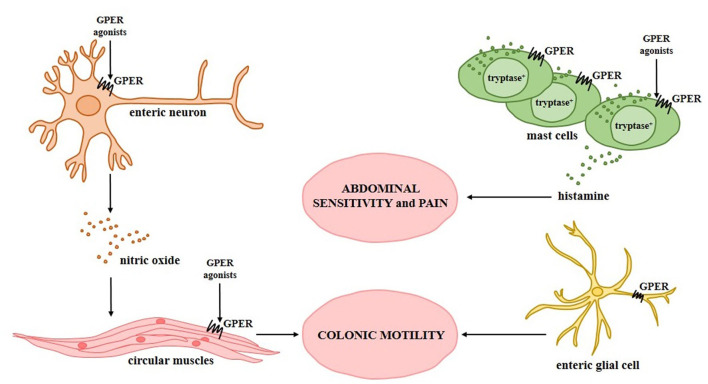
Relationship between G protein-coupled estrogen receptor and irritable bowel syndrome. Experimentally confirmed GPER activity in specific types of intestinal cells during the progression of irritable bowel syndrome and its association with the symptoms observed in patients with irritable bowel syndrome is shown.

Potential mechanisms by which GPER can modulate progression of irritable bowel syndrome are summarized in Figure 2.

## Inflammatory Bowel Diseases

Crohn's disease (CD) and ulcerative colitis (UC) are the major types of inflammatory bowel disease (IBDs) that manifest themselves as chronic intestine inflammation. The most evidence emphasizing estrogen significance in IBDs comes from clinical observations that take into account hormone fluctuations in pre-menopausal and post-menopausal women in the prevalence and symptoms of IBDs (34–36). On the other hand, many reports indicate that estrogen receptors regulate the immune response affecting not only intestinal cells, but also immune cells (37–41). However, the importance of G protein-coupled estrogen receptor in IBDs is still poorly understood, but available data indicate that GPER may be involved in the pathways responsible for progression of IBDs.

It was documented that patients with CD and UC are characterized by a lack of changes, both in the level of circulating 17β-estradiol and enzymes involved in estrogen metabolism in relation to reference values and control group. Nevertheless, alterations in estrogen receptor expression, including GPER were found in the intestine of patients with IBDs (42–44). Significantly higher levels of GPER in the intestine have been demonstrated in both CD and UC patients compared to healthy controls. Higher expression of GPER at the mRNA level was also observed in an independent cohort using dataset provided by Gene Expression Omnibus. Nevertheless, when sex and age of women were taken into consideration, higher expression of GPER at the protein level was found in the intestine of men with both types of IBDs in relation to sex-matched controls. In the case of women, up-regulation of GPER expression in the intestine of women with UC under the age of 50 compared to sex and age-matched controls was noted (43). Interesting evidence was also provided by Włodarczyk et al. (42) who documented differences in GPER expression in non-inflamed and inflamed intestine obtained from patients with CD, but not in patients with UC. Alterations in GPER expression in IBDs suggest that GPER may be involved in the immune response in the progression of colitis in patients with CD and UC.

**Figure 3:**
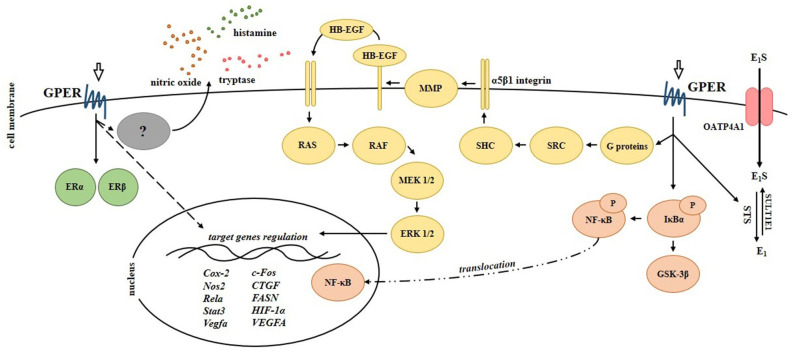
Signaling pathways regulated by G protein-coupled estrogen receptor in the intestinal cells. Scheme shows experimentally proven signals mediated by GPER in bowel diseases. Cox-2, cycloxygenase-2; CTGF, connective tissue growth factor; E1, estrone; E_1_S, estrone sulfate; ERα, estrogen receptor α; ERβ, estrogen receptor β; EGFR, epidermal growth factor receptor; ERK 1/2, extracellular signal-regulated kinase 1/2; FASN, fatty acid synthase; c-Fos, FBJ osteosarcoma (subunit of AP1 transcription factor); GSK-3β, glycogen synthase kinase-3β; HB-EGF, heparin-binding epidermal growth factor; HIF-1α, hypoxia-inducible factor-1α; IκBα, NF-κB inhibitor α; MEK 1/2, mitogen-activated protein kinase kinase; MMP, matrix metalloproteinase; NF-κB, nuclear factor κ-light-chain-enhancer of activated B cells; Nos2, nitric oxide synthase 2; OATP4A1, organic anion transporter polypeptide 4A1; P, phosphorylation; RAF, rapidly accelerated fibrosarcoma (serine-threonine kinase); RAS, rat sarcoma (small GTPase); Rela, nuclear factor NF-κB subunit; SHC, adapter protein containing SRC homology 2 domain; SRC, non-receptor tyrosine kinase; Stat3, signal transducer and activator of transcription 3; STS, steroid sulfatase; SULT1E1, sulfotransferase family 1E member 1; Vegfa/VEGFA, vascular endothelial growth factor A.

The functional significance of GPER in CD was provided by *in vivo* studies using trinitrobenzene sulfonic acid (TNBS)-induced CD model in mice (44, 45). It was demonstrated that modulation of GPER activity using estrogen receptor agonists and antagonists affects the development of colitis. GPER activation has been shown to improve macroscopic and microscopic scores as well as reduces the mortality of mice with CD in relation to untreated mice with CD. In contrast, inhibition of GPER by its selective antagonist G15, was found to be not associated with an improvement of the above mentioned parameters and mortality in the murine model of CD. It is worth noting that GPER is overexpressed in the intestine of male mice with induced CD, as in the intestine of men with CD (43, 44). Interestingly, GPER agonists and antagonists affect not only GPER but also nuclear ERs expression and localization. Immunohistochemical analysis revealed that ERα is localized in the cytoplasm of goblet cells in the intestine of control and TNBS-treated mice supplemented with G-1 and 17β-estradiol. In contrast, the lack of ERα expression in the cytoplasm of goblet cells has been documented in the intestine of TNBS-treated and TNBS-treated mice supplemented with GPER selective antagonist. Accumulating body of evidence suggests that estrogen signaling is strictly synchronized and all estrogen receptors play a crucial role in the regulation of several major cellular signaling pathways (20, 46–52). In the intestine cross-talk between estrogen receptors seems to be important of patients with CD and the value of ERα/ERβ ratio in the serum may be useful to predict endoscopic activity in CD patients (53). At the molecular level GPER appears to be engaged in the modulation of signaling pathway of extracellular signal-regulated kinases (ERKs), which leads to changes in expression of immune-related genes which was documented in the intestine of IBDs patients and murine model. It was shown that intestine of mice with CD is characterized by higher expression of immunomodulatory genes and GPER activation significantly reduces the levels of immune-related genes (44). The major signaling pathways induced by GPER in bowel diseases are summarized in Figure 3.

## Colorectal Cancer

Current evidence based on experimental studies and available datasets provided by Gene Expression Omnibus and Oncomine documented lower expression of GPER at the mRNA and protein levels in the intestine of colorectal cancer (CRC) patients compared to adjacent control tissue (11). Moreover, a gradual decrease of GPER expression appears to be associated with CRC stage and lymph node metastasis in CRC patients (11). Liu et al. (11) provided evidence indicating that promoter methylation and histone H3 deacetylation represent mechanisms responsible for regulation of GPER expression in CRC. Conflicting results regarding the clinical relevance of GPER expression in CRC patients are from Kaplan-Meier analysis (11, 54, 55). Lower expression of GPER in the intestine of CRC patients seems to be associated with poorer survival rate in relation to CRC patients with high intestinal expression of GPER (11). On the other hand, Bustos et al. (54) reported that the survival is affected by the sexual dimorphism of CRC patients. Higher expression of GPER has been shown to be associated with poor relapse-free survival in women with stage 3 and 4 but not stage 1 and 2 CRC or men regardless of stage.

The first studies highlighting the role of estrogen signaling through GPER in CRC were conducted by Santolla et al. (56) who found a link between GPER and fatty acid synthase (FASN) in the neoplastic transformation of colon. FASN is a key lipogenic enzyme which is able to act as a metabolic oncogene in several types of cancer, such as breast and colorectal cancer (57–59). Functional cross-talk between GPER activation, FASN expression and CRC cells proliferation and migration has been documented. *In vitro* studies using GPER agonists and LoVo cells have revealed that GPER by affecting the EGFR/ERK/c-Fos/AP1 signaling pathway is responsible for regulation of FASN expression and modulates the potential for CRC cells proliferation and migration (56). In studies covering a wide spectrum of potential mechanisms that may be involved in CRC progression, Liu et al. (11) found that GPER participates in numerous processes and pathways affecting proliferation of CRC cells. Both *in vitro* and *in vivo* studies revealed that GPER is engaged in cell cycle, endoplasmic reticulum stress and modulation of apoptosis which are crucial in the regulation of proliferation, migration and invasion of CRC cells. Liu et al. (11) reported that CRC cells treated with GPER selective agonist G-1 have a higher proportion of cells in the apoptotic phase and their mitochondria are characterized by lower membrane polarity. In line, protein expression analysis showed that treatment of CRC cells with G-1 is related with up-regulation of pro-apoptotic while down-regulation of anti-apoptotic proteins. Activation of reactive oxygen species, ERKs signal and NF-κB suppression appear to be involved in the inhibition of GPER-mediated CRC cell growth. Two-way action of GPER depending on the oxygen level in CRC cells was suggested by Bustos et al. (54). GPER has been shown to be able to modulate two major angiogenic factors, i.e., hypoxia inducible factor (HIF) and vascular endothelial growth factor (VEGF), which are related to the progression of many cancer types. It was found that, under normoxic condition GPER mediates inhibition whereas under hypoxic conditions GPER enhances HIF-1α and VEGFA expression in CRC cells. Beyond regulation of hypoxia-related genes, estrogens acting through GPER seem to potentiate hypoxia-induced proliferation and migration of CRC cells, while under normoxic condition they suppress cell proliferation and migration of CRC cells (54).

Studies conducted by Gilligan et al. (55, 60) clarify the impact of local concentrations of active estrogens and subsequent action on the development and progression of CRC. Clinical observations have indicated that local steroid sulfatase (STS) and 17β-hydroxysteroid dehydrogenase (HSD17) B2, B7, and B12 activity and expression in the intestine of CRC patients are disturbed, favoring 17β-estradiol synthesis in the intestine of CRC patients. STS is an enzyme which converts circulating sulfated estrogen into active form, while HSD17B7 and HSD17B12 catalyze the conversion of estrone to 17β-estradiol and HSD17B2 catalyzes the conversion of 17β-estradiol to estrone (61). Both 17β-estradiol administration and STS overexpression are related with increased CRC cell proliferation, which has been confirmed in *in vitro* and *in vivo* models (55, 60). Interestingly, estrogens increase proliferation through GPER signaling affecting expression of connective tissue growth factor (CTGF), which is crucial factor related to the proliferation, survival, and migration of cancer cells (55). On the other hand, modulation of GPER activity affects STS activity and may act as an estrogenic positive feedback loop leading to the development and progression of CRC (60).

Despite the inconclusive results regarding the level of GPER expression and activity in CRC, the membrane-bound estrogen receptor seems to be an important factor responsible for the modulation of multiple processes leading to the development and progression of CRC. The reasons for these discrepancies may result, for example, from differences in study groups in terms of gender and age. In above mentioned studies, it was proved that the patient's sex seems to be critical in assessing the role of the G protein-coupled estrogen receptor in intestinal diseases. Additionally, the hormonal status of women (pre- and post-menopausal period) is not insignificant and should be taken into consideration in studies on estrogen signaling in intestinal diseases. Different effects of GPER may also be the result of specific cellular context. As demonstrated by Bustos et al. (54) in order to fully understand an estrogenic response it is essential to appreciate not only the estrogen receptor status of the tumor cells but also the hypoxic conditions of the local tumor microenvironment. However, further analysis using clinical material and *in vivo* models are needed to determine significance of GPER in CRC.

## Conclusions

**Figure 4:**
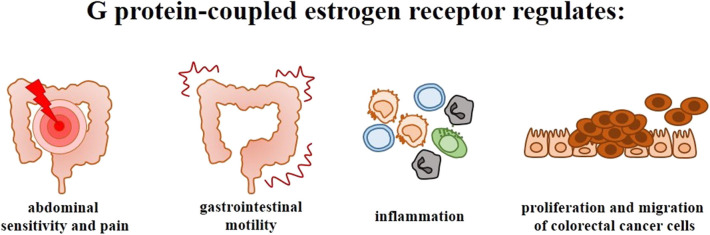
Significance of G protein-coupled estrogen receptor in the intestinal diseases. Scheme illustrating the main symptoms and phenomena regulated by GPER in irritable bowel syndrome, inflammatory bowel diseases and colorectal cancer.

Clinical and experimental studies indicate that nuclear ERs, especially ERβ which is mainly expressed in the intestine, play an important role in the proliferation and differentiation of intestine cells as well as in the organization and maintenance of intestine architecture. Nevertheless, the available findings support the thesis, that in addition to nuclear ERs, GPER expression and activity is responsible for the development and progression of intestinal diseases, i.e., irritable bowel syndrome, inflammatory bowel diseases and colorectal cancer. GPER has been shown to be involved in abdominal sensitivity and pain, intestinal motility, and colitis as well as proliferation and migration of colorectal cancer cells (Figure 4). However, further studies are needed to determine clinical and therapeutic potential of GPER in bowel diseases.

## Author Contributions

DJ and WK contributed to writing, critical revisions, editing and approval of the submitted version of manuscript.

## Conflict of Interest

The authors declare that the research was conducted in the absence of any commercial or financial relationships that could be construed as a potential conflict of interest.
